# In Vivo Antimalarial Effects of Iranian Flora *Artemisia khorassanica* against *Plasmodium berghei* and Pharmacochemistry of its Natural Components

**Published:** 2010-03

**Authors:** H Nahrevanian, B Esmaeili, M Kazemi, H Nazem, M Amini

**Affiliations:** 1 Department of Parasitology, Pasteur Institute of Iran, Tehran, Iran; 2 Payame Nour University, Tehran Center, Tehran, Iran; 3 Department of Applied Chemistry, Islamic Azad University, Qom Branch, Iran

**Keywords:** *Artemisia khorassanica*, Iran, Malaria, Pharmacochemistry, *Plasmodium berghei*

## Abstract

**Background:**

The aim of this study was to evaluate the antimalarial effects of Iranian flora *Artemisia khorassanica* against *Plasmodium berghei* *in vivo* and pharmacochemistry of its natural components.

**Methods:**

The aerial parts of Iranian flora *A. khorasanica* were collected at flowering stage from Khorassan Province, northeastern Iran in 2008. They were air-dried at room temperature; powder was macerated in methanol and the extract defatted in refrigerator, filtered, diluted with water, then eluted with n-hexane and finally non-polar components were identified through Gas Chromatography and Mass Spectroscopy (GC-MS). Toxicity of herbal extracts was assessed on naïve NMRI mice, and its anti-malarial efficacy was investigated on infected *Plasmodium berghei* animals. This is the first application on *A. khorssanica* extract for treatment of murine malaria. The significance of differences was determined by Analysis of Variances (ANOVA) and Student's *t*-test using Graph Pad Prism Software.

**Results:**

The herbal extract was successfully tested *in vivo* for its anti-plasmodial activity through artemisin composition, which is widely used as a standard malaria treatment.

**Conclusion:**

Although, this study confirmed less anti-malarial effects of *A. khorssanica* against murine malaria *in vivo*, however there are some evidences on reducing pathophysiology by this medication. In complementary assay, major components were detected by GC-MS analysis in herbal extract including chrysanthenone (7.8%), palmitic acid (7.4%) and cis-thujone (5.8%). The most retention indices of the component are given as n-eicosane, palmitic acid and n-octadecane.

## Introduction

Malaria, is one of the most serious and widespread diseases encountered by human. It is an infectious disease caused by the parasite *Plasmodia (P.)* transmitted by the female anopheles mosquitoes. Four identified species of this parasite exist, which cause different types of human malaria (1). Although all the four species of malaria parasites can infect humans and cause illness, only *P. falciparum* is known to be potentially life threatening and some of infected persons die, usually because of delayed treatment (2). An annual incidence of 300-500 million clinically cases and 1-2 million death occur in the world (3–6).

As malaria vaccines remain problematic, chemotherapy still is the most important weapon in the fight against the disease (7). The antimalarial drugs including chloroquine, quinine, mefloquine, pyrimethamine and artemisinine are currently used to prevent and treat human malaria. Part of the reason for the failure to control malaria, is the spread of resistance to first-line antimalarial drugs, cross-resistance between the limited number of drug families available, and some multidrug resistance (8). Resistance has emerged to all classes of antimalarial drugs except artemisinin, an endoperoxide antimalarial drug derived as the active component of *Artemisia annua*, a herbal remedy used in Chinese folk medicine for 2000 years "qinghaosu" (9–12). Artemisinin is a powerful anti-malarial drug with significant activities, which is resistant to chloroquine. It is a natural product, which has the characteristics of high potency against the parasite whilst possessing low toxicity during treatment of malaria infections (13–15).

The genus *Artemisia* has always been of great pharmaceutical interest and is useful in traditional medicines for a treatment of the variety of diseases (11, 16, 17). *A. annua* is presently being cultivated on a commercial scale in China and Vietnam for its antimalarial sesquiterpene lactone. The genus is of small herbs found in Northern temperate regions and belongs to the important family Compositae (Asteraceae), one of the most bulky vegetal groupings, which comprises about1000 genera and over 20,000 species. Within this family, *Artemisia* is included into the family Anthemideae and comprises itself over 400 species, found in Europe and North America, but mainly are dominating the Asia (18–20). Among the Asian *Artemisia* flora, 150 species were recorded for China, 50 species reported to occur in Japan and 34 species of the genus are found in Iran, of which may be endemic; *A. melanolepis Boiss* and *A. kermanensis Pold* (21), *A. absinthium* (22), *A. annua* (23), *A. dracunculus* (24), A*. aucheri* (25), *A. haussknechtii Boiss* (26), *A. scoparia, A. sieberi* (27) and *A. sieberi Besser* (28).

Pharmacochemical analysis of artemisinin shows that the structure of this compound is rather unique among natural products as it contains the very unusual 1, 2, 4-trioxane ring system. It was sufficiently unusual that it was originally characterized as an ozonide until revised crystallographic analysis provided unambiguous structural elucidation (29–33).

For a drug to be effective against the malaria parasite, it must reach the site of action in sufficient concentration and then interact with the receptors before it is either deactivated and/or eliminated by the host or the parasite. Extensive pharmacological and biochemical evaluation revealed that this compound was a blood schizonticide preferentially imported into malaria-infected erythrocytes via the parasitophorous duct (34). It has been also shown that artemisinin and related trioxanes demonstrate useful activity against selected carcinomas (35). Due to complex chemical structure of artemisinin, the chemical synthesis of the molecule is complex, which results in very low yields and the cost becomes prohibitory to use synthetic approach for its commercial production (36).

The mechanism of the action of artemisinin remains a mystery, although iron appears to be involved in activating this endoperoxide to generate cytotoxic free radicals (37). Several candidates have been hypothesized as targets of artemisinins, including haem and some parasite membrane proteins (37–39), however; none of these has been convincingly shown to be functionally relevant. Recently, some researcher proposed *Pf*ATP6, as artemisinin's target (40); however, this conclusion has also been debated (41).

For a better understanding of specific fractions with high efficacy and low side effects, chemical analysis of genus of Iranian *A. khorassanica* was required. Pharmacochemistry and chemical analysis of different genus of Iranian *Artemisia* species has been studied and the presence of variety of components including monoterpenes (42), sesquiterpenes (43, 44), sesquiterpene lactones (42, 45, 46) and essential oils (47–51) were fully reported (22–28). This genus is uniform and the chemistry is somewhat diverse. However, most species contain sesquiterpene lactones, especially 11,13- dihydro derivatives (42–51).

The aim of this study was to evaluate Iranian flora *A. khorassanica* for its antimalarial effects against *Plasmodium berghei* *in vivo* and pharmacochemical analysis of its natural components. This is the first report on *A. khorasanica* extract as flora from the Khorassan Province, northeastern Iran for treatment of murine malaria on *P. berghei* infected NMRI mice. The major components were also detected by Gas Chromatography and Mass Spectroscopy (GC-MS) analysis.

## Materials and Methods

### Plant samples

The aerial parts of Iranian flora *A. khorasanica* were collected at flowering stage from their natural habitats the Shahroud Mountains in Khorassan Province, northeastern Iran in 2008. Voucher specimens were deposited and identified at the Herbarium of the Research Institute of Forests and Rangelands (RIFR), Tehran, Iran.

### Extraction of Herbal Extract and Non-polar Compounds

The aerial parts were air-dried at room temperature and were then powdered. The herbs powder (650 g) of A. *khorassanica* was macerated in methanol (1 lit) and subsequently kept for 48-72 h. It was then filtered and evaporated at reduced pressure to give a crude extract (50 ml). The extract was defatted at -15°C in refrigerator, filtered, added with water (20% w/w) and then eluted with n-hexane (50 ml). Finally, n-hexane phase was collected, evaporated by rotary evaporator (20 ml) and then non-polar components were identified through GC-MS analysis (52).

Gas Chromatography (GC): GC analysis was performed on a Shimadzu 15A gas chromatograph equipped with a spilt/spilt less (ratio 1:30), injector (250°C) and a flame ionization detector (250°C). N_2_ was used as carrier gas (1 mL/min) and the capillary column used was DB-5 (50 m × 0.2 mm, film thickness 0.32 pin). The column temperature was kept at 60°C for 3 min and then heated to 220°C with a 5°C/min rate and kept constant at 220°C for 5 min. Relative percentage amounts were calculated from peak area using a Shimadzu C-R4A chromatopac without the use of correction factors (53).

GC/Mass Spectrometry (MS): GC/MS analysis was performed using a Hewlett-Packard (HP-6890) with a HP-5MS column (30 m × 0.25 mm, film thickness 0.25 µm). The column temperature was kept at 60°C for 3 min, programmed to 220°C at a rate of 5°C/min, and kept constant at 220°C for 5 min. The flow rate of Helium as carrier gas was (1 mL/min). MS were taken at 70 eV, mass rang, 30 to 350 amu and scan time, 2 scan/ sec (53).

Identification of components: The components of the oil were identified by comparison of their mass spectra with those of a computer library or with authentic compounds and confirmed by comparison of their retention indices either with those of authentic compounds or with data published in the literature (54). The retention indices were calculated for all volatile constituents using a homologous series of n-alkanes (53).

### Animals

Male outbred NMRI (Naval Medical Research Institute) mice (supplied by the Laboratory Animal Department, Karaj Production, and Research Complex, Pasteur Institute of Iran) were used in this study. The mice were housed at room temperature (20–23°C) on a 12 h light and 12 h dark cycle, with unlimited access to food and tap water.

### Ethical considerations

Experiments with animals were done according to the ethical standards formulated in the Declaration of Helsinki, and measures taken to protect animals from pain or discomfort. It has been approved by institutional ethical review board (Ethical Committee of the Pasteur Institute of Iran), in which the antimalarial test was done.

### Malaria parasite

*P. berghei* NY was kindly donated by Dr. M. J. Dascombe from the School of Life Sciences, University of Manchester, UK. Malaria parasite was maintained by blood passage in NMRI mice when active parasites were required; otherwise it was stored at -70°C in Alserver's solution (2.33% glucose, 0.525% NaCl and 1% sodium citrate in deionised water) and glycerol (9:1 parts by volume) (54).

### Inoculation of malaria parasites

Mice were inoculated (0.2 ml) intravenously (iv) into a tail vein with blood from a donor mouse (38% parasitaemia *P. berghei*) diluted with 0.85% saline to contain 2×10^6^ parasitised red blood cells (PRBC).

#### Experiments and groups

**A) Toxicity assay of A. khorassanica herbal extract in naïve animals**

In vivo toxicity was assessed by using herbal extract on naïve NMRI male mice. Animals were divided into four groups (n=8 mice/group), including Group 1 (naïve), Group 2 (low dose), Group 3 (average dose), Group 4 (high dose). A sample of herbal extract were suspended in ethanol and normal saline (1:9), then three different concentrations (low, average and high doses) of herbal extracts including 1, 10 and 100 mg/ml were tested *in vivo* for its toxicity as test animals and a control group which was injected with drug vehicle. Entire animals in all groups were injected with 200 µl of related solutions subcutaneously (sc) once a day for 8 days.

**B) Anti-malarial effects of herbal extract on P. berghei infected mice**

Following toxicity assay, the highest dose with the lowest toxicity of herbal extract (100 mg/ml concentration) was selected to apply for its antimalarial activity on male NMRI mice infected with *P. berghei*. Animals were divided into two groups (n=10 mice/group), including control and test; both groups were infected with murine malaria parasite, *P. berghei*. Drug vehicle and herbal extract was injected sc into control and test groups respectively once a day with 200 µl of solutions for the period of three weeks.

### Assessment of pathology

#### Parasitaemia

The clinical diagnosis was confirmed by laboratory demonstration of the malaria parasite in the stained smears. In all animals, parasitaemia was determined on different days after infection using blood smears stained with Geimsa stain (Sigma Chemical Co., USA). PRBC were counted in five different fields, each of approximately 200 cells. Results are expressed as the mean percentage (%) of erythrocytes containing Geimsa bodies. Experiments were licensed under the Animals (Scientific Procedures) Act 1986. In compliance with the conditions of this license, infected animals were humanely killed at the onset of the terminal phase of malaria (*P. berghei*) infection (54, 55).

### Assessment of degree of hepato/ splenomegaly

Entire livers and spleens were removed post mortem at the end of the experimental period from mice after induction of terminal general anaesthesia by inhalation of diethyl ether (Sigma Co., Germany). Organ wet weights were measured and compared with controls as indices for degree of hepatomegaly and splenomegaly (56, 57).

### Body weight

Body weight was measured initially and at different times of experiment (days 1, 7, 21) using a top pan balance (OHAUS Scale Corp., USA) as a major indication of pathology (56, 67).

### Statistical analysis

Values are presented as the mean±SEM for groups of *n* mice. The significance of differences was determined by Analysis of Variances (ANOVA) and Student's *t*-test using Graph Pad Prism Software (GraphPad, San Diego, California, USA).

## Results

**Fig. 1:**
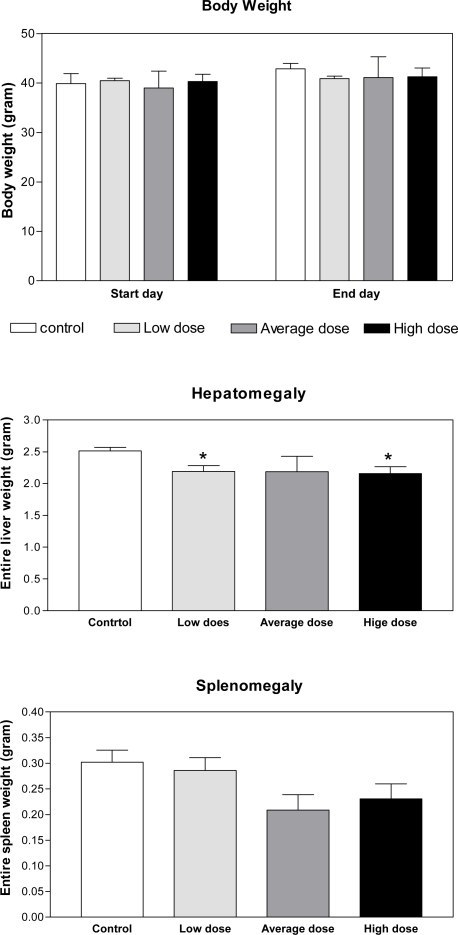
Toxicity assay and pathophysiological changes induced by *A. khorassanica* crude extract in naive animals Pathophysiological changes including body weight, hepatomegaly and splenomegaly were evaluated in control and test groups as toxicity assay induced by injection of low, average and high doses of *A. khorassanica* crude extract (n=8, *P<0.05, ANOVA)

The results of toxicity assay in naïve mice indicated no significant pathophysiological changes in body weight and splenomegaly in test groups as compared with those in control after injection of low, average and high doses of *A. khorassanica* crude extracts. There was a small reduction (*P*<0.05) in hepatomegaly of test groups injected with low and high doses of herbal crude extract, which emphasises the anti- symptomatic effects of *A. khorassanica* (Fig. 1).

**Fig. 2:**
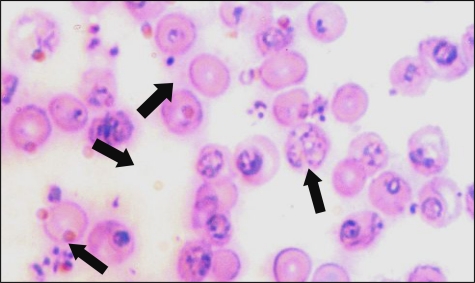
*Plasmodium berghei* blood-stage forms in Geimsa stained smears from mice. The clinical diagnosis was confirmed by laboratory demonstration of the malaria parasite in the stained smears. Parasitaemia was determined using blood smears stained with Geimsa stain. PRBC were counted in five different fields, each of approximately 200 cells. Results are expressed as the mean percentage of erythrocytes containing Geimsa positive bodies

**Fig. 3:**
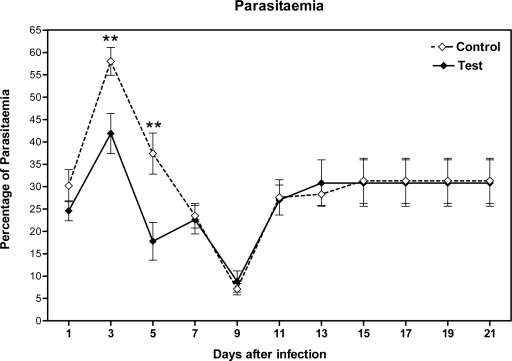
Percentage of parasitaemia in smears from blood of malarial mice Smears were dried in air, fixed by methanol and stained with Geimsa for counting of parasites inside RBC by light microscopy, Test, *A. khorassanica* crude extract; Control, Drug vehicle (n=10 mice/day/group, Student's *t*-test, **P<0.01)

The clinical diagnosis of *P. berghei* infection was confirmed by laboratory demonstration of the malaria parasite in the stained smears. Parasitaemia was determined using blood smears stained with Geimsa stain from mice (Fig. 2). The observations specifically indicated the inhibitory effects of the *A. khorassanica* extracts on the early developmental stages of *P. berghei* by decreasing parasitaemia (*P*<0.01). This may suggest that the active constituents in the herbal extract may be toxic for *P. berghei*, thereby inhibiting their development to the erythrocytic stage (Fig. 3).

**Fig. 4:**
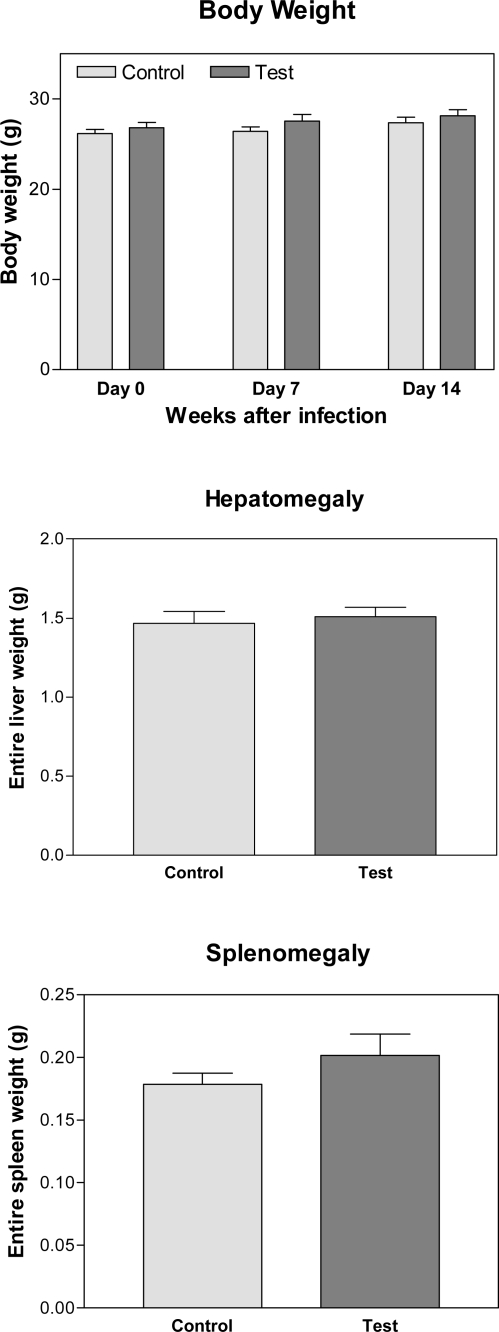
Toxicity assay and pathophysiological changes induced by *A. khorassanica* crude extract in malarial animals. Pathophysiological changes including body weight, hepatomegaly and splenomegaly were evaluated as indices of toxicity by crude extract of *A. khorassanica* in control and malarial groups (n=10 mice/day/group, Student's *t*-test)

No pathophysiological changes including body weight, hepatomegaly and splenomegaly were detected in control and malarial groups as induction markers for toxicity after injection of crude extract of *A. khorassanica* in malarial infected animals (Fig. 4).

The chemical analysis of extract by GC-MS on a HP-6890 instrument resulted in isolation of 31 fractions and identification of effective components. The non-polar components obtained from extract *A. khorassanica* are listed in Table 1, in which the percentage and retention indices of the component are given. Nineteen compounds, representing 53.7% of the total constituents in the non-polar compounds of *A. khorassanica* were characterized by chrisanthenone (7.8%) and palmitic acid (7.4%). Monoterpenes constitute the major fraction of the oil (29.9%), while sesquiterpenes and other compounds accounted to 6.2% and 17.6% respectively.

**Table 1:** The non-polar constituents obtained from extract of *A. khorassanica* by GC-MS. The non-polar constituents obtained from *A. khorassanica* were assessed by GC-MS and listed in this table, in which the percentage and retention indices of the component are given.

Compound	Retention indices (RI)	*A. khorassanica* (%W/W)
n-nonane	899	0.6
dihydromyrcene	947	0.5
4-methyl nonane	958	0.6
n-decane	999	2.1
1,8-cineole*	1033	4.0
cis-thujone	1102	5.8
trans-thujone*	1114	3.7
isophorone	1118	2.7
chrysanthenone	1123	7.8
champhore	1143	5.4
n-dodecane	1199	1.1
n-tetradecane	1398	1.5
davanone	1586	2.8
n-hexadecane*	1600	1.7
beta-davanone-2-ol*	1717	3.4
n-octadecane*	1800	1.4
palmitic acid*	1984	7.4
n-eicosane*	2000	1.2

## Discussion

Malaria is one of the most serious and widespread diseases and as malaria vaccines remain problematic, chemotherapy still is the most important weapon against the disease. However, the increasing drug resistance continues to be the main problem, therefore, the limited clinical repertoire of effective drugs and the emergence of multi-resistant strains substantiate the need for new anti-malarials (7).

The results of this study indicated no toxicity in naïve mice with even high dose of *A. khorassanica* crude extracts, which confirms its lowest side effects. Although, the inhibitory effects of the *A. khorassanica* extract on the early decline of *P. berghei* parasitaemia highlights its anti-malarial activity, however this concept no longer can be observed in the late infection. This may be due to the metabolic process of *A. khorassanica* crude extract by mice and reduction of its concentration in body. Malaria parasite actually decreases body weight and increases hepatomegaly and splenomegaly. Crude extract of *A. khorassanica* represented its anti-symptomatic effects by stabilization of body, liver and spleen weights.

In this study, 31 fractions were isolated from *A. khorssanica* extract and effective components were identified by the chemical analysis of extract by GC-MS. The highest percentages of the components are indicated as chrysanthenone (7.8%), palmitic acid (7.4%) and cis-thujone (5.8%). The most retention indices of the component are given as n-eicosane, palmitic acid and n-octadecane. In other studies, various species of the genus *Artemisia* are used for their pharmacological, antimicrobial, antioxidant activity. Three species of this genus, *A. scoparia*, *A. sieberi* and *A. aucheri* are widely distributed in desert area of Iran (27). This is the first report on application of *A. khorssanica* extract on the treatment of murine malaria. The herbal extract was successfully tested *in vivo* for its anti-malarial activity through artemisin composition, which is widely used as a standard malaria therapy. Although, this study confirmed anti-malarial effects of *A. khorssanica* extracts against murine malaria *in vivo* during early infection, however there are more efficacies on pathophysiological symptoms by this medication. These observations provide the basis for the traditional use of this herb in treatments of malaria disease (58).

The route of inoculation is important factor to determine herbal efficacy. Although, subcutaneous injection was used in this study, other routes may be recommended for future studies. Moreover, active derivatives of *Artemisia* including artemether, arteether and artesunate, which are used for oral, intramuscular, rectal and intravenous administration (59). More investigations on different *Plasmodia* and animal hosts are needed to better clarify anti-malarial activity of Iranian flora *A. khorassanica* and analysis of its natural components.
